# Impact of pre-travel consultation on clinical management and outcomes of travelers’ diarrhea: a retrospective cohort study

**DOI:** 10.1186/s40794-018-0076-2

**Published:** 2018-12-04

**Authors:** Eugene M. Tan, Jennifer L. St. Sauver, Irene G. Sia

**Affiliations:** 1 Division of Infectious Diseases, Department of Medicine, Mayo Clinic, 200 1st St SW, Rochester, MN 55905 USA; 2 Division of Epidemiology, Center for the Science of Health Care Delivery, Mayo Clinic, 200 1st St SW, Rochester, MN 55905 USA

**Keywords:** Travelers’ diarrhea, Pre-travel consultation, Infectious disease

## Abstract

**Background:**

International travelers are at high risk of acquiring travelers’ diarrhea. Pre-travel consultation has been associated with lower rates of malaria, hepatitis, and human immunodeficiency virus (HIV) infections. The objective was to study the impact of pre-travel consultation on clinical management and outcomes of travelers’ diarrhea.

**Methods:**

This retrospective cohort study analyzed 1160 patients diagnosed with travelers’ diarrhea at Mayo Clinic Rochester, MN from 1994 to 2017. Variables included high-risk activities, post-travel care utilization, antimicrobial prescriptions, hospitalizations, and complications. Travelers were divided into those who sought (*n* = 256) and did not seek (*n* = 904) pre-travel consultation. The two groups were compared using the Wilcoxon test for continuous variables and chi-square test for categorical variables. Multivariate logistic regression was used to adjust for differences in traveler characteristics.

**Results:**

More pre-travel consultation recipients were young Caucasians who had more post-travel infectious disease (ID) consultation [OR 3.1 (95% CI 1.9–5.3)], more stool sampling [OR 1.6 (95% CI 1.1–2.4)], and more antimicrobial prescriptions [OR 1.6 (95% CI 1.1–2.5)] for travelers’ diarrhea compared to the non-pre-travel consultation group. The pre-travel consultation group had shorter hospital stays (mean 1.8 days for pre-travel versus 3.3 days for non-pre-travel consultation group, *p* = 0.006) and reduced gastroenterology consultation rates [OR 0.4 (95% CI 0.2–0.9)]. 23 patients with positive stool cultures had *Campylobacter* susceptibilities performed; 65% (15/23) demonstrated intermediate susceptibility or resistance to ciprofloxacin.

**Conclusion:**

Pre-travel consultation was associated with higher rates of stool testing and antimicrobial prescriptions. The high rate of quinolone-resistant *Campylobacter* in our small sample suggests the need for judicious antimicrobial utilization. The pre-travel consultation group did have a shorter duration of hospitalization and reduced need for gastroenterology consultation for prolonged or severe symptoms, which are positive outcomes that reflect reduced morbidity of travelers’ diarrhea.

## Background

In 2015, there were 1.2 billion international arrivals to the United States, [1] and of those, 60 million were undertaken by American citizens [2]. International travelers are at high risk of acquiring travel-related infections such as travelers’ diarrhea [3]. Pre-travel consultation may benefit travelers by educating them on a variety of topics such as food and water precautions, destination-specific vaccinations, and travelers’ diarrhea self-management [4]. In a EuroTravNet study from 2008 to 2012, pre-travel consultation was associated with less malaria, hepatitis, and human immunodeficiency virus (HIV) infections [5].

Travelers’ diarrhea is particularly important because it is so common and can affect approximately 40% of returning travelers [3]. Although pre-travel consultation may reduce the rate of malaria infection, it may not reduce the rate of travelers’ diarrhea. There is no proven effective preventive measure or vaccine for travelers’ diarrhea, which is why it continues to affect such a large proportion of travelers [5].

The objective of this study was to examine the impact of pre-travel consultation on clinical management and outcomes of travelers’ diarrhea. The hypothesis was that patients with travelers’ diarrhea who received pre-travel consultation would have improved clinical outcomes compared to those without pre-travel consultation. Specific aims included comparing high-risk travel behaviors as they relate to food and water consumption, itineraries, and hospitalization and complication rates for travelers with and without pre-travel consultation.

## Methods

### Study design, period, and area

This single-center retrospective cohort study included patients of all ages who received medical care for travelers’ diarrhea at Mayo Clinic Rochester, MN, USA, between January 1, 1994, and December 31, 2016. Travelers’ diarrhea was defined as the passage of three or more unformed stools in a 24-h period within 10 days of return from international travel [6]. Persons that granted permission for their medical records to be used for research (Minnesota Research Authorization) were included in the study, which was approved by the Mayo Clinic Institutional Review Board.

All patients with travelers’ diarrhea were identified through the Advanced Cohort Explorer (ACE), which is an institutional search engine that located 7025 charts containing various spellings and capitalizations of the keyword *travelers’ diarrhea*. We also searched for “infectious gastroenteritis and colitis, unspecified” (ICD-10 diagnosis code A09); “diarrhea, unspecified” (ICD-10 diagnosis code R19.7); “enterotoxigenic *Escherichia coli* infection” (ICD-10 diagnosis code A04.1); and “infectious diarrhea” (ICD-9 diagnosis code 009.2). As these diagnosis codes were not specific for travelers’ diarrhea, we then searched for keywords referencing international travel. Charts were manually reviewed and excluded if the keyword *travelers’ diarrhea* was stated only in the context of pre-travel consultation, not for post-travel illness. Patients identified as having travelers’ diarrhea associated with domestic travel only were excluded. These inclusion and exclusion criteria yielded the final sample size of 1160 patients with travelers’ diarrhea.

These patients were subdivided into those who had (*n* = 256) and those who did not have (*n* = 904) pre-travel consultation. Pre-travel consultation is provided by the Travel and Tropical Medicine Clinic (TTMC) within the Division of Infectious Diseases (ID) at Mayo Clinic. Out of the 256 patients in the pre-travel consultation group, 210 received their consultation at the Mayo TTMC, and the remaining 46 received their consultation through a primary care provider. Given the relatively small number (*n* = 46) of patients who sought pre-travel consultation outside the Mayo TTMC, they were included in the same group as the Mayo pre-travel consultation recipients for statistical analysis.

### Demographics

**Fig. 1:**
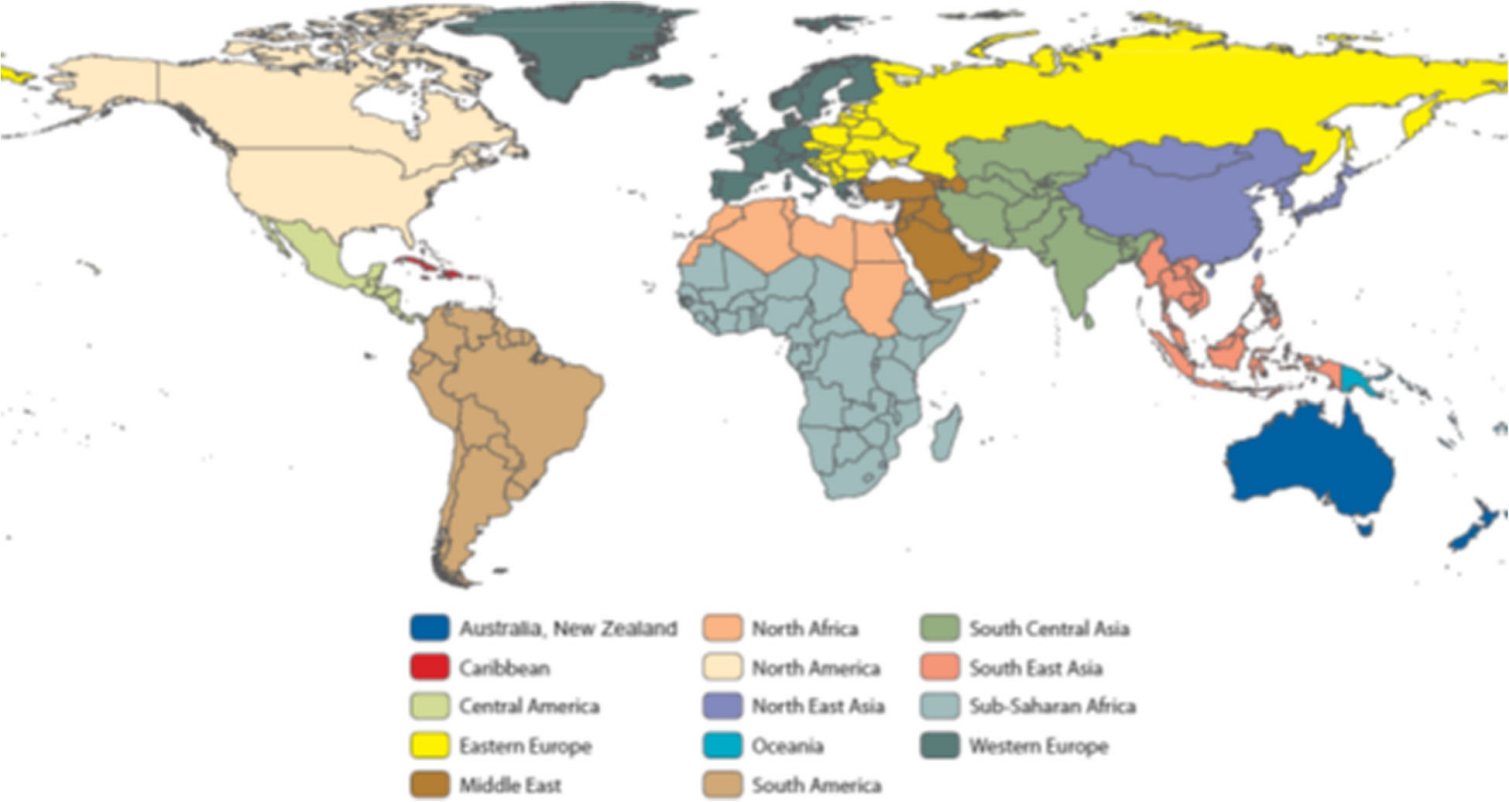
Geographic region of exposure based on GeoSentinel Surveillance System [7] (All material in the Morbidity and Mortality Weekly Report (MMWR) Series is in the public domain and may be used and reprinted without permission)

Demographic information included age, gender, ethnicity, employment, and local residence (i.e., residing in Olmsted County). Destinations of travel were recorded based on the classifications provided by the GeoSentinel Surveillance System (Fig. 1) [7]. These categories were not mutually exclusive because travelers often visited multiple destinations.

### Travel itineraries

Information on itineraries included travel reason, destination, and duration. High-risk travel was defined as any activity that may increase the risk of food/waterborne illness, such as consuming unsanitary food (e.g. undercooked meat, unwashed fruits, or salads) or drinking tap water, traveling to a rural area without ready access to health care, and camping or hiking in a remote area where there may not be access to potable water or hygiene facilities.

### Pre-travel counseling utilization

Pre-travel consultation at the TTMC follows a standardized protocol that includes education on safe food and water consumption, avoidance of high-risk travel activities, recommendations for appropriate vaccines, and provision of antidiarrheal antibiotics for self-treatment. Vaccine recommendations were based on the travel itinerary and patient’s medical and immunization history, following Centers for Disease Control and Prevention (CDC) guidelines [8]. International travelers to developing countries often are advised to receive the hepatitis A and typhoid fever vaccines. Preventive medication prescriptions included antibiotics (azithromycin or quinolones) for presumptive treatment of travelers’ diarrhea. For those who did seek pre-travel consultation, prescription rates of vaccines and antidiarrheal antibiotics were recorded. In addition to the index episode of travel, utilization of pre-travel consultation for later and past episodes of travel was recorded. A portion of pre-travel consultation recipients (18%, 46/256) received their consultation outside the Mayo TTMC; if these outside consultation notes documented similar discussion points as our Mayo TTMC notes, these patients were deemed as having completed pre-travel consultation.

### Travelers’ diarrhea: Clinical management and outcomes

After returning with travelers’ diarrhea, patients presented for medical care in various settings: non-visit care (i.e. telephone or patient online service correspondence), primary care, emergency or urgent care, ID, or other subspecialty clinic. In the course of the post-travel medical evaluation, stool samples may be obtained for testing, and the microbiology of infection was recorded if documented.

As our study period ranged from 1994 to 2017, there were changes in diagnostic testing during this long timeframe. Though conventional stool culture was the sole method of testing for many years, our institution implemented a limited bacterial enteric pathogen polymerase chain reaction (PCR) panel on August 23, 2010, which tested for only *Campylobacter*, *Shigella*, *E. coli*, *Yersinia*, and *Salmonella.* Clinicians had the option of ordering either the stool culture or the limited PCR panel, and the choice of either test varied widely depending on the individual providers.

On October 12, 2015, our institution implemented the Gastrointestinal FilmArray® Panel by BioFire, which is a multiplex PCR panel that detects twenty-two common bacterial, parasitic, and viral gastrointestinal pathogens. As part of the new laboratory testing algorithm, stool culture would only be performed if the BioFire panel was negative and if diarrhea persisted. The type of stool sampling (culture versus PCR) and the results of any susceptibility panels were recorded, if available.

Clinical management and outcomes were assessed through the following variables: antimicrobial prescriptions, development of *Clostridium difficile* infection after antimicrobial use, hospitalization rate and duration, short-term complications (e.g. dehydration or acute kidney injury), long-term complications (chronic diarrhea lasting over four weeks), and need for gastroenterology consultation.

### Data analysis

Descriptive statistics were used to determine if traveler characteristics, management of diarrhea, and diarrhea outcomes differed between patients who did and did not utilize pre-travel consultation. Primary measures were compared between the pre-travel and non-pre-travel consultation groups using the Wilcoxon test for continuous variables and chi-square test for categorical variables. Multivariate logistic regression was used to adjust for differences in traveler characteristics. JMP® 13 Pro was used for statistical analysis.

## Results

### Demographics

Characteristics of patients with travelers’ diarrhea who did and did not utilize pre-travel consultation are shown in Table 1. Patients who utilized pre-travel consultation were younger and more likely to be students. There was a high proportion of Caucasians in both pre-travel and non-pre-travel consultation groups (Table 1).

**Table 1:** Demographics of patients with travelers’ diarrhea (*n* = 1160)

	Pre-travel consultation^a^		*p* value^b^
	Yes (*n* = 256)	No (n = 904)	
Age, in years	35 (23–53)	43 (27–56)	0.0001
Male	107 (42)	413 (46)	0.3
Employed	153 (60)	626 (69)	0.0004
Unemployed	103 (40)	278 (31)	
*Student*	*64 (25)*	*102 (11)*	
*Retired*	*16 (6)*	*106 (12)*	
*Other*	*23 (9)*	*70 (8)*	
Caucasian	208 (81)	794 (88)	0.007
Local resident^c^	177 (69)	529 (59)	0.002
Traveler sought pre-travel consult for *future* travel	92 (36)^2^	116 (13)	< 0.0001
Traveler sought pre-travel consult for *prior* travel	60 (23)	116 (13)	< 0.0001

^a^Continuous variables are expressed as median (interquartile range). IQR = interquartile range from the 25th percentile to the 75th percentile. Categorical variables are expressed as numbers (%)

^b^*P* values were calculated based on Pearson’s chi-square test for categorical variables and Wilcoxon’s rank sum test for continuous variables

^c^Local residents were defined as those whose primary residence was in Olmsted County, Minnesota, at the time of travel

### Travel itineraries

The major destination for travelers returning with diarrhea was Central America, specifically Mexico, of whom the majority (565/904, 63%) did not seek pre-travel consultation. In contrast, for more distant destinations such as South America, Sub-Saharan Africa, and South Asia, more patients with travelers’ diarrhea did seek pre-travel consultation (Table 2).

**Table 2:** Travel destinations based on geographic regions, as defined by the GeoSentinel Surveillance Network, for patients with travelers’ diarrhea (*n* = 1160). Travelers often frequented multiple destinations

Region	Pre-travel consultation^a^		*p* value^b^
	Yes (*n* = 256)	No (*n* = 904)	
Central America	55 (22)	565 (63)	< 0.0001
*Mexico*	*17 (7)*	*494 (55)*	
South America	36 (14)	51 (6)	< 0.0001
Sub-Saharan Africa	65 (25)	20 (2)	< 0.0001
South Asia	42 (16)	32 (4)	< 0.0001
Southeast Asia	36 (14)	31 (3)	< 0.0001
Northeast Asia	20 (8)	31 (3)	0.0080
Caribbean	23 (9)	117 (13)	0.2

^a^Categorical variables are expressed as numbers (%)

^b^*P* values were calculated based on Pearson’s chi-square test for categorical variables

A greater percentage of vacation travelers did not seek pre-travel consultation, whereas a higher percentage of business, volunteer, and visiting friends and relatives (VFR) travelers sought pre-travel consultation (Table 3). Information on duration of travel was available for only 47% (542/1160) patients. Those who sought pre-travel consultation traveled for longer durations, traveling almost twice as long as those who did not seek pre-travel consultation. Patients with travelers’ diarrhea reported various high-risk activities. There were higher percentages of all high-risk activities, such as unsanitary food and water consumption, in the pre-travel versus the non-pre-travel consultation group (Table 3).

**Table 3:** Travel characteristics

	Pre-travel consultation^a^		*p* value^b^
	Yes (*n* = 256)	No (*n* = 904)	
Days of travel^c^	15 (9–32)	8 (7–14)	< 0.0001
Reasons:			
Vacation	83 (32)	379 (42)	0.006
Business	54 (21)	55 (6)	< 0.0001
Volunteer work	68 (27)	33 (4)	< 0.0001
VFR^d^	38 (15)	61 (7)	< 0.0001
Unknown	13 (5)	376 (42)	< 0.0001
Unsanitary food	65 (24)	189 (21)	0.07
Unsanitary water	39 (15)	104 (12)	0.0003
Rural location	137 (54)	39 (4)	< 0.0001
Hiking	42 (16)	15 (2)	< 0.0001
Camping	25 (10)	2 (0.2)	< 0.0001

^a^Categorical variables are expressed as numbers (%)

^b^*P* values were calculated based on Pearson’s chi-square test for categorical variables and Wilcoxon’s rank sum test for continuous variables

^c^Duration of travel was recorded as a continuous variable, which was expressed as median (interquartile range). IQR = interquartile range from the 25th percentile to the 75th percentile. Information on duration of travel was available for only 47% (542/1160) patients

^d^VFRs are immigrants or the children of immigrants, who are from developing nations and return home to visit friends and relatives

### Pre-travel counseling utilization

About three-quarters (78%) of those reporting travelers’ diarrhea did not receive pre-travel consultation. Of those patients who received pre-travel consultation, a majority (220/256, 86%) received prescriptions for antidiarrheal antibiotics before departure. These pre-travel antibiotic prescriptions included quinolones (144/256, 66%) and azithromycin (76/256, 35%). Depending on destination-specific risk factors for disease, hepatitis A and typhoid fever vaccines were recommended to most, but not all, travelers who sought pre-travel consultation. The pre-travel consultation group had a higher rate of hepatitis A vaccine completion [53% (134/255)] compared to the non-pre-travel consultation group [20% (177/868), *p* < 0.0001]. The pre-travel consultation group also had a higher rate of typhoid fever vaccine completion: [72% (169/234)] compared to the non-pre-travel consultation group [5% (41/839), *p* < 0.0001]. Patients who sought pre-travel consultation prior to the index episode of travelers’ diarrhea had higher rates of both future and past pre-travel consultations, compared to the non-pre-travel consultation group (Table 1).

### Travelers’ diarrhea: Clinical management and outcomes

After returning from travel (Table 4), patients who did not have pre-travel consultation presented to primary care providers (39%) more than those who did (29%, *p* < 0.0001). Those who sought pre-travel consultation had higher rates of post-travel specialty ID consultation (34% in pre-travel consultation group versus 9% in non-pre-travel consultation group, *p* < 0.0001). Non-visit care, such as telephone or patient online correspondence, was a common way for patients in both groups to seek medical advice.

**Table 4:** Clinical management and outcomes of travelers’ diarrhea

	Pre-travel consultation^a^		*p* value^b^
	Yes (*n* = 256)	No (*n* = 904)	
1st health care encounter			
Primary Care	73 (29)	348 (39)	
Infectious Disease (ID)	39 (15)	38 (4)	< 0.0001
Emergency/Urgent Care	32 (13)	137 (15)	
Other Specialty^c^	13 (5)	89 (10)	
Non-Visit Care^d^	99 (39)	292 (32)	
ID consultation	86 (34)	82 (9)	< 0.0001
Stool sample	134 (52)	331 (37)	< 0.0001
Pathogens found	34/134 (25)	80/331 (24)	0.8
Microbiology:			
*Campylobacter*	10/34 (29)	28/80 (35)	0.6
*Escherichia coli*	5/34 (15)	17/80 (21)	0.4
*Salmonella*	5/34 (15)	17/80 (21)	0.4
*Shigella*	2/34 (6)	3/80 (4)	0.6
*Giardia*	6/34 (18)	6/80 (8)	0.1
Post-travel antimicrobial prescribed^e^	187 (73)	563 (62)	0.002
*Clostridium difficile* infection after antimicrobials	4/187 (2)	9/563 (2)	0.6
Hospitalization	17 (7)	53 (6)	0.6
Number of hospital days	1 (1–2)	2 (1–4)	0.02
Short-term complications	32 (13)	100 (11)	0.5
Diarrhea lasting > 4 weeks	50 (20)	155 (17)	0.4
*Gastroenterology consultation*	*13/50 (26)*	*85/155 (55)*	*0.0004*

^a^Categorical variables are expressed as numbers (%). Continuous variables are expressed as median (interquartile range). *IQR* interquartile range from the 25th percentile to the 75th percentile

^b^*P* values were calculated based on Pearson’s chi-square test for categorical variables

^c^Other specialties included gastroenterology, endocrinology, cardiology, transplant, surgery, etc

^d^Non-visit care included telephone calls or patient online correspondence between patients and providers

^e^Post-travel antimicrobials were prescribed either empirically or based on microbiology results, if available. Common post-travel antimicrobials included azithromycin and ciprofloxacin

A greater percentage (52%) of the pre-travel consultation group had stool samples sent for microbiologic testing, compared to the non-pre-travel consultation group (37%, *p* < 0.0001). Of those who had stool testing done in both groups, *Campylobacter* species (38/114, 33%) were the most common bacterial pathogens, followed by *Escherichia coli* (22/114, 19%) and *Salmonella* species (22/114, 19%).

When diagnosed with travelers’ diarrhea, a greater percentage (73%) of the pre-travel consultation group had antimicrobials prescribed, compared to the non-pre-travel consultation group (62%, *p* = 0.002). Overall, a small proportion of individuals in both groups (6–7%) required hospital admission. Though there was no significant difference in rates of hospitalization between the two groups, the duration of hospitalization was shorter in the pre-travel consultation group (median 1 day) compared to the non-pre-travel consultation group (median 2 days, *p* = 0.02). There were no significant differences in short-term complications, which included dehydration and acute kidney injury, nor in chronic diarrhea (lasting > 4 weeks) between the two groups. Of those with chronic diarrhea, more patients in the non-pre-travel consultation group (55%) saw a gastroenterology specialist compared to the pre-travel consultation group (26%, *p* = 0.0004).

Table 5 shows the results of multivariate analyses performed to adjust for differences in baseline traveler characteristics, such as age, sex, race, travel destination, and high-risk behaviors. The fully adjusted multivariate model showed that the pre-travel consultation group had more ID consultation, less gastroenterology consultation, more stool samples obtained, more antimicrobials prescribed, and shorter hospital stay.

**Table 5:** Multivariate analysis for clinical management and outcome variables

	Pre-travel consultation (compared to no pre-travel consultation)^a^		
	Unadjusted odds ratio (95% confidence interval)	Partially adjusted odds ratio (95% confidence interval)^b^	Fully adjusted odds ratio (95% confidence interval)^c^
Infectious Disease Consult	5.1 (3.6–7.2)	5.4 (3.8–7.7)	3.1 (1.9–5.3)
Gastroenterology Consult	0.3 (0.1–0.6)	0.2 (0.1–0.5)	0.4 (0.2–0.9)
Mean Days of Hospitalization	*p* = 0.009	*p* = 0.06	p = 0.006
Pre-travel consult	1.8 days	1.8 days	1.8 days
No pre-travel consult	3.3 days	3.3 days	3.3 days
Stool sample obtained	1.9 (1.4–2.5)	1.9 (1.4–2.5)	1.6 (1.1–2.4)
Antimicrobial prescribed	1.6 (1.2–2.2)	1.7 (1.2–2.3)	1.6 (1.1–2.5)

^a^A nominal logistic model was used for categorical variables. Least squares means were used to estimate the mean hospitalization duration based on a linear model. *P* values were calculated based on least squares means

^b^Partially adjusted model included age, sex, and race

^c^Fully adjusted model included age, sex; race; local residence; employment; travel destinations (Central America, South America, Sub-Saharan Africa, South Asia, Southeast Asia, Northeast Asia, Caribbean); unsanitary food and water consumption; rural location; hiking; camping

### Drug resistance

Stool pathogens were isolated from 114 patients with travelers’ diarrhea in both the pre-travel and non-pre-travel counseling groups. *Campylobacter* species were detected in 38 (33%) samples: 87% (33/38) were isolated through stool culture, whereas the remaining 13% (5/38) were detected through polymerase chain reaction (PCR) only. Susceptibility testing on 23/33 (70%) on the isolates from stool cultures showed 65% (15/23) of *Campylobacter* isolates with intermediate susceptibility or resistance to ciprofloxacin, as defined by a minimum inhibitory concentration greater than or equal to 2 μg/mL [9].

Among this small sample (*n* = 15) of patients with ciprofloxacin-resistant *Campylobacter* species, the majority traveled to Mexico (6, 40%); other destinations included Dominican Republic (2, 14%); Belize, Tanzania, Venezuela, Colombia, Bolivia, Peru, and Jamaica (each with 1 case, 7%). All of these patients with quinolone-resistant *Campylobacter* species had resolution of their diarrhea. 67% (10/15) were treated with azithromycin, based on susceptibility results. Interestingly, despite quinolone resistance, 13% (2/15) experienced resolution of symptoms with ciprofloxacin treatment, and 20% (3/15) experienced resolution with no antimicrobials at all.

## Discussion

Pre-travel consultation recipients had increased rates of post-travel ID consultation, stool testing, and antimicrobial prescriptions for travelers’ diarrhea. However, the pre-travel consultation group had a reduced duration of hospitalization and need for subsequent gastroenterology consultation for prolonged or severe symptoms; this may have reduced the overall long-term morbidity of disease.

### Demographics

The pre-travel consultation group was largely characterized by young Caucasians. The younger age range may be explained by the greater proportion of students in the pre-travel consultation group. Often, students who travel for educational, volunteer, or religious purposes are required by their school or organization to seek pre-travel consultation. The large number of patients in this study who did not seek pre-travel consultation is consistent with the previous finding that up to 80% of travelers do not seek pre-travel consultation [10]. This suggests the need for additional education for potential travelers on the utility of pre-travel consultation, particularly when traveling to destinations that carry a high risk for acquiring travel-related illnesses. The pre-travel consultation group’s higher rates of past and later pre-travel consultations attests to the value that these patients obtained from these visits.

### Travel itineraries

The most popular travel destination was Central America, specifically Mexico, for which many travelers did not seek pre-travel consultation; this may be associated with the greater proportion of vacation travelers. For farther destinations such as South America, Sub-Saharan Africa, and South Asia, a greater proportion of travelers did seek pre-travel consultation, which may be associated with the greater proportion of business and volunteer work travelers. Though the reason for this discrepancy in pre-travel consultation rates among vacation, business, and volunteer work travelers is unknown, these findings are consistent with a past study on pediatric travel consultation, in which patients seen in travel clinic were more likely to travel for humanitarian work or parental work relocation, whereas vacation travelers were more likely to be seen in a general medical clinic [11].

The hypothesis was that the pre-travel consultation group would demonstrate lower rates of participation in high-risk activities, such as unsanitary food and water consumption. Paradoxically, however, the opposite association was found. It is possible that these risk factors were not modifiable in the pre-travel consultation group, as this group did plan longer, more distant, more rural, and potentially higher-risk itineraries. There may simply not have been easy access to clean food or water available in some of the destinations that the pre-travel consultation group frequented. This finding is consistent with past observations, which have shown that rates of travelers’ diarrhea have not changed significantly over a 50-year period despite pre-travel counseling. Travelers may have a limited ability to select places serving food based on hygiene, and pre-travel advice regarding avoidance of certain foods may be too difficult and impractical to follow [12]. Another possibility is that the pre-travel consultation group truly did engage in higher-risk activities, as the provision of a “safety net” of antidiarrheal medications prescribed during their pre-travel consultation may have subconsciously created a sense of complacency in this group.

### Travelers’ diarrhea: Clinical management and outcomes

Pre-travel consultation at Mayo Clinic is offered by the Division of Infectious Diseases (ID). Therefore, those travelers who seek pre-travel consultation are connected to care with the ID division and may be more likely to seek ID consultation for post-travel illness or future travel-related medical issues. This finding is reflected in Table 1, which shows that a greater proportion of patients with travelers’ diarrhea and pre-travel consultation utilized ID services post-travel. This specific demographic may have demonstrated more health care-seeking behavior at baseline, possibly due to more comprehensive insurance plans. However, as insurance expenses and out-of-pocket costs were not measured, it is difficult to draw a specific conclusion.

The association of ID consultation with favorable clinical outcomes supports a potentially important role of the ID physician in the management of travelers’ diarrhea. Patients in the pre-travel consultation group had improved outcomes compared to the non-pre-travel consultation group. For example, the pre-travel consultation group had a shorter duration of hospitalization. Although there was a statistically significant decrease in duration of hospitalization, the relatively small sample size and the median difference of only one day may not make this a clinically significant result.

Stool testing may facilitate appropriate diagnosis and management when patients return with severe symptoms. According to the 2017 Infectious Diseases Society of America (IDSA) Clinical Practice Guidelines for the Diagnosis and Management of Infectious Diarrhea, stool testing should be performed for travelers to resource-challenged areas if they have severe or persistent symptoms or fail empiric therapy [13, 14].

Though the pre-travel consultation group had a shorter duration of hospitalization and less need for gastroenterology consultation for prolonged or severe symptoms, it is uncertain if these positive outcomes were associated with the higher rate of ID consultation, stool testing, and antimicrobial prescription. ID specialists may be ordering an excessive number of stool testing and antimicrobials for patients in whom symptoms would resolve naturally. Though most patients with quinolone-resistant *Campylobacter* species experienced resolution of symptoms with azithromycin in our study, some did not require antimicrobials at all, and some improved with ciprofloxacin, suggesting the self-resolving nature of the disease. Nonetheless, although travelers’ diarrhea may be self-limited and resolve without antibiotics in most cases, there is overall strong evidence for the effectiveness of antimicrobials in most patients with moderate to severe travelers’ diarrhea [13, 14]. According to a Cochrane Review in 2000, antimicrobials can effectively decrease the overall duration of illness by 48–72 h [15].

### Drug resistance

*Campylobacter* species in Southeast and South Asia are known to have widespread resistance to fluoroquinolones [14]. However, the prevalence of quinolone resistance is still higher in other regions of the world (e.g. 65% in Southern Europe, 60% in the Middle East, and 40% in Africa) [16]. Though limited by a small sample size, our study showed a quinolone resistance rate of 65% (15/23), which occurred mostly among travelers to Latin America and the Caribbean. This underscores the need for antimicrobial stewardship, as the routine use of quinolones for travelers’ diarrhea may be ineffective.

### Limitations

The major limitations of this study include its retrospective nature, missing data, and lack of a healthy control group. It is known that 20–80% of travelers do not seek pre-travel consultation [10]. As many of these travelers may not become ill nor seek medical care, it is impossible to know the actual number of travelers in our population; hence, we do not know the true burden of disease from travelers’ diarrhea. We do not have data on how many of those with antibiotic prescriptions for travelers’ diarrhea self-treatment took the antibiotic for diarrheal episodes that occurred during their travel. The truly interesting question is, “How many episodes of travelers’ diarrhea are prevented through pre-travel consultation?” Though intriguing and clinically useful, this question may be impossible to answer as it is difficult to prove that pre-travel interventions lead to the absence of disease.

## Conclusions

Pre-travel consultation was associated with higher rates of stool testing and antimicrobial prescriptions for travelers’ diarrhea. Antimicrobials have not shown any benefit in preventing long-term sequelae of travelers’ diarrhea, such as irritable bowel syndrome, reactive arthritis, and Guillain–Barré syndrome [14]. Given the increase in drug resistance among bacterial pathogens, adopting a formal antimicrobial stewardship policy regarding the post-travel treatment of diarrhea would be prudent. However, the pre-travel consultation group had a shorter duration of hospitalization and reduced need for gastroenterology consultation, which are undoubtedly positive outcomes that reflect reduced morbidity of disease. Travelers who sought pre-travel consultation had improved rates of hepatitis A and typhoid fever vaccine completion. Pre-travel consultation may be a prime opportunity to educate patients on ways to prevent travel-associated gastrointestinal infections, including hepatitis A and typhoid fever vaccinations and appropriate hand hygiene. Further study on patients’ perceptions of pre-travel services may provide insight on the impact of pre-travel consultation on post-travel illness.

## Abbreviations

CI: confidence interval
ID: infectious diseases
OR: odds ratio
TTMC: Travel and Tropical Medicine Clinic
